# A qualitative study of the experiences of insulin use by older people with type 2 diabetes mellitus

**DOI:** 10.1186/s12875-024-02318-3

**Published:** 2024-05-22

**Authors:** Chaya Langerman, Angus Forbes, Glenn Robert

**Affiliations:** Florence Nightingale Faculty of Nursing, Midwifery and Palliative Care, King’s College London, London, UK

**Keywords:** Experiences, Insulin, Older people, Type 2 diabetes mellitus

## Abstract

**Background:**

There is a rising prevalence of type 2 diabetes among older people. This population also suffers from co-morbidity and a greater number of diabetes related complications, such as visual and cognitive impairment, which can potentially affect their ability to manage insulin regimens. Understanding the experiences of older people when they transition to insulin will help the development of healthcare interventions to enhance their diabetes outcomes, overall health and quality of life.

**Aims:**

The aims of this exploratory study were to (1) understand the experiences of older people with type 2 diabetes in relation to insulin treatment initiation and management and (2) use this understanding to consider how the insulin management support provided to older people by healthcare providers could be more tailored to their needs.

**Method:**

A qualitative study using semi structured (remote) interviews with older people with diabetes (*n* = 10) and caregivers (*n* = 4) from the UK. Interviews were audio recorded and transcribed, and framework analysis was used to analyse the data.

**Results:**

Three main themes, along with six subthemes, were generated from the study data. Participants generally felt at ease with insulin administration following training, yet some reported feelings of failure at transitioning to insulin use. Participants were also frustrated at what they perceived were insufficient resources for effective self-management, coupled with a lack of professional interest in optimising their health as older people. Some also expressed dissatisfaction regarding the brevity of their consultations, inconsistent information from different healthcare professionals and poor treatment coordination between primary and secondary care.

**Conclusion:**

Overall, the study emphasised that older people need better support, education and resources to help manage their insulin use. Healthcare professionals should be encouraged to adopt a more individualised approach to supporting older people that acknowledges their prior knowledge, physical and psychological capabilities and motivation for diabetes self-management. In addition, better communication between different services and greater access to specialist support is clearly needed for this older population.

**Practice implications:**

An integrated care pathway for insulin use in older people could be considered. This would include an assessment of the older person’s needs and capacity on their initiation to insulin; targeted education and training in self-management; timely access to appropriate emotional and peer support resources; care plans developed collaboratively with patients; and individualised glucose targets that recognise the needs and preferences of the older person.

**Supplementary Information:**

The online version contains supplementary material available at 10.1186/s12875-024-02318-3.

## Introduction

Current global estimates for diabetes show that half of all adults diagnosed with type 2 diabetes (T2DM) are over 65 years of age [1]. Diabetes is a progressive disease, and as endogenous insulin secretion declines, a significant proportion of patients will require insulin to manage their blood glucose [2, 3]. Consequently, many older people living with diabetes need to use insulin to avoid excessively high glucose levels. However, insulin use in older people can bring additional hazards such as hypoglycaemia [4, 5] which can increase the risk of falls, cardiovascular morbidity, hospital admissions and mortality [6, 7]. Preventing hypoglycaemia in older people, therefore, is an important consideration when introducing insulin therapy. Older people also face challenges in terms of poor vision, dexterity, and memory issues which can hamper their appropriate administration of insulin treatment. Equally, common comorbidities in older people, such as arthritis and depression [8], can cause further difficulties with self-care. Relatedly, concerns regarding the capacity of older people to administer insulin and accurately self-monitor blood glucose can lead professionals to delay insulin initiation in this population [9].

There is limited empirical literature that explores the issues that older people face with transitioning to and managing insulin treatment. A recent meta-synthesis which focused on the use of insulin by older people with diabetes reported that it was often complicated by difficulties with cognition, dexterity and comorbidity [10]. However, the synthesis of insulin use found very few studies that had focused solely on older people, whilst studies with wider age ranges generally failed to provide sub-group analyses of people over 65 years. In addition, none of the studies that focused specifically on insulin use in older populations had been conducted within the last five years [11, 12]. This is problematic as older studies are unlikely to reflect changes in diabetes treatment, for example the insulin types (human and analogue) or the delivery systems (syringe vs. pen) available. In order to develop services or interventions which effectively support insulin use, it is paramount that research features current technology. It is also important to give this older population, who are often neglected in research studies, a chance to provide their lived experiences [13]. Previous research which has focused on insulin use in older people has been limited to quantitative methods. Adopting a qualitative approach helps to deepen our understanding of both the context and impact of age-related challenges to managing insulin in this population [14].

The exploratory study reported in this paper therefore aimed to (1) better understand the experiences of older people with T2DM in relation to insulin treatment initiation and ongoing management and (2) to use this understanding to consider how the insulin management support provided to older people by healthcare providers could be more tailored to their needs.

## Methods

Semi structured narrative interviews were held with people with T2DM and carers of older people with T2DM to explore their experiences of using insulin therapy. Older people on insulin are commonly supported by family members. Therefore, a decision was made to also include informal carers, who can provide a unique perspective on the daily challenges of insulin therapy for older people. The study reporting adhered to the COREQ guidelines [15].

All participants were recruited remotely in the UK by advertising on the websites of the voluntary organisation Diabetes UK (DUK) and the research networks of the National Institute of Health and Care Research (NIHR). Recruiting from the support groups of DUK, which is a national patient organisation, ensured that the research opportunity was advertised across a diverse range of geographical regions. The advertisement invited potential participants (older people and carers) to contact the researcher directly to express their interest.

All older people who agreed to participate were asked whether they would like to invite their carers to take part in the study. Carers who indicated interest were also encouraged to invite their older relative to participate. However, all eligible participants were accepted into the study, regardless of whether their family member was interested in participating. Older people and carers from the same family could choose whether they wanted to be interviewed together or separately to ensure that all participants felt comfortable during the interview.

### Study context

This study involved people with T2DM receiving treatment within the National Health Service (NHS) in the UK. In the NHS, T2DM is managed in primary rather than specialist care settings, hence, most patients with T2DM using insulin are managed by their general practitioner (GP) and/or practice nurse, although a few with more complex needs may be seen by community specialist diabetes services [16]. Patients who are initiated on to insulin will receive information by healthcare professionals on the practicalities of insulin administration, but there is no standard structured education provided following initiation and subsequent care can be ad hoc [17]. People are encouraged to monitor their blood glucose as part of their self-care. However, in the UK at the time of conducting the study, continuous glucose monitoring (CGM) and flash glucose monitoring were exclusively provided within the NHS to people with type 1 diabetes, they were generally not available to people with T2DM.

### Study eligibility

The eligibility criteria for older people were as follows:

- Being aged 70 years or over and diagnosed with T2DM.
- Being on insulin treatment for 6–48 months (the lower limit was set to ensure adequate experience of insulin and the upper limit to avoid difficulty with longer-term recall regarding initial experiences of insulin).
- Having sufficient English language skills to be interviewed.
- Having access to appropriate technology to take part in an online interview.

Eligibility for informal carers was as follows:

- Aged over eighteen years.
- Providing care to an older person with T2DM who had been on insulin for 6–48 months.
- Having sufficient English language skills to be interviewed.
- Having access to appropriate technology to take part in an online interview.

We chose seventy years and above as our eligibility criterion in order to align with the definition of the European Diabetes Working Party for Older People 2011 Clinical Guidelines for T2DM [18]. Participants were sent an information pack and given the opportunity to talk about the research with the lead author (CL). The lead author ensured that all participants who took part fully understood the aims of the research and could give informed consent. Written consent to participate in this study was obtained from all participants before any research was conducted. Interviews were conducted online using MS Teams due to restrictions arising from the COVID-19 pandemic. Participants were given a £20 shopping voucher for their involvement. Ethical approval for the study was obtained from the Psychiatry, Nursing and Midwifery Research Ethics Subcommittee, King’s College London (reference LRS/DP-21/22-27077).

Our data collection method, sampling strategy, analysis and interpretations followed the Elo et al. [19] checklist to improve the trustworthiness of this study. Semi structured narrative interviews were considered the most appropriate way to collect rich data on the individual experiences of older people [20]. While there is no established optimal size for qualitative studies [21] the concept of information power has been proposed as a pragmatic approach to sampling [22]. Information power relates the adequacy of a sample size to the specificity of the study’s aim, the sample diversity and the quality of data collected. This study had a specific aim of exploring experiences of insulin use among a relatively homogeneous group of older participants with T2DM through interviews with an experienced researcher. Considering this, a sample size of between 12 and 15 interviewees is appropriate and aligns with previous recommendations for qualitative research [21].

Participants were given the flexibility to select their preferred interview format from virtual, telephone, or in-person options. Participants were provided with comprehensive guidance and support to facilitate their use of the online platform. The researcher also dedicated time to assisting participants in becoming familiar with the technology to ensure a smooth interview process. The online interviews were conducted by one researcher (CL). Older people and carer interviews followed the same topic guide, with minor edits to phrasing to ensure questions made sense to each group (supplement 1.) Pilot interviews and focus groups had been conducted previously to inform the study protocol and the topic guide. The interviews aimed to elicit the experience of older people and carers with regards to insulin initiation and ongoing management. In order to encourage meaningful conversations, the researcher probed when necessary to address research questions whilst being careful not to dominate the interview. Data was examined consecutively after each interview was conducted for close data monitoring. All interviews were conducted between September 2021 and November 2022.

## Data analysis

The interviews were digitally recorded, transcribed verbatim and imported into NVivo version 10 for analysis. Framework analysis was chosen to analyse the data for its flexibility, highly structured process and the fact that it is untied to a particular theoretical stance [23]. To ensure credibility, all members of the research team were involved in the analysis. Themes were derived iteratively from the data, rather than through previously defined concepts, and followed a five-step approach:

1. Familiarisation: one author (CL) repeatedly read the transcripts to become familiar with the data set.
2. Identifying a thematic framework: the same author (CL) coded the entire data set and a second author (GR) independently coded a subset of transcripts. These two coders then met with a third author (AF) to discuss discrepancies between the two sets and generate an initial thematic framework.
3. Coding/indexing: the framework was systematically and independently applied to the transcripts by CL, GR and AF, who met frequently to discuss coding application and reach consensus where needed.
4. Charting: framework matrices were created in NVivo for each theme to which the data were entered.
5. Mapping and interpretation: the data were transferred to a table for each theme. Data were grouped, and key dimensions, which became the main themes of the results, were identified.

The research team ensured reflexivity throughout the process by being mindful of how their professional and academic backgrounds might influence data collection and analysis. GR and CL are academic researchers with an interest in codesign and user experiences of healthcare services. AF is an academic and a specialist clinical nurse with experience of treating people with diabetes within the NHS.

## Results

Fourteen older people contacted the researcher, and eleven initially consented to be interviewed. However, one participant subsequently withdrew, resulting in a total sample of ten older people. Seven of the fourteen carers who contacted the researcher consented but two later decided not to participate and one could not be reached, resulting in four carer interviews. Only one of the carers recruited was a relative of an older person participant and they chose to be interviewed separately. The remaining carer participants who were interviewed did not have relatives who were taking part in the study. All interviews lasted between 45 and 60 min.

Table 1. details the participant characteristics and shows the diversity of geographic location, cohabitation status, gender distribution and duration of insulin usage. The mean patient age (years) was 72.6 (± 2.15) and the mean duration on insulin (months) was 21.3 (± 11.86). Two of the older people interviewed reported that they were dependent on an informal carer for support.

**Table 1:** Participant characteristics

No	Patient/Carer (P/C)	Age (Years)	Gender (F/M)	Ethnicity (white/non-white)	Duration of insulin (Months)	Cohabitation Status (Y/N informal carer)	Relationship with Patient	Geographic region
1	P	70	M	W	24	Y		Southwest
2	P	72	M	NW	6	N		Southeast
3	P	70	M	NW	36	Y		Southeast
4	P	72	F	NW	32	Y		Southeast
5	P	72	M	NW	24	Y		Northwest
6	P	75	M	NW	12	Y		West Midlands
7	C	28	F	-	-	-	Daughter in law	West Midlands
8	P	75	M	NW	6	N		Southeast
9	C	42	F	-	-	-	daughter	Southeast
10	P	70	M	W	9	Y		Scotland
11	C	NA	F	-	-	-	Family member	Southwest
12	P	76	F	W	40	Y		North
13	P	74	F	W	24	N		East
14	C	NA	F	-	-	-	daughter	Southwest

*Carer-dependent.

**Fig. 1:**
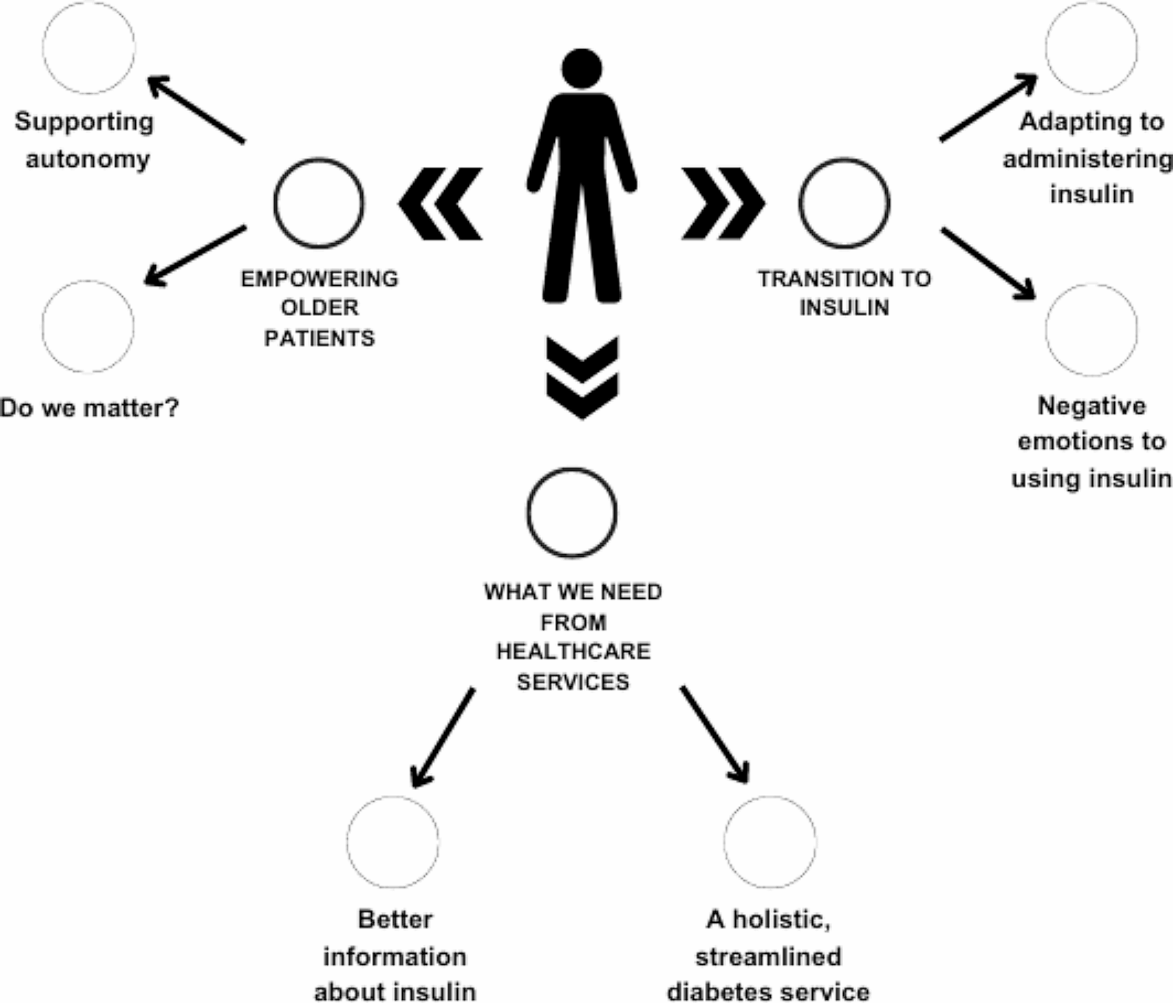
Identified themes and subthemes

Three main themes were identified which comprised six subthemes (Fig. 1):

Theme 1: The transition to insulin (subthemes: adapting to administering insulin, negative emotions connected with insulin use); Theme 2: What we need from a service (subthemes: better information about insulin, a holistic streamlined service); and Theme 3: Empowering older people (subthemes: supporting autonomy, do we matter?). These themes are described in turn below together with illustrative quotations from the interview data.

### Theme 1: transition to insulin

#### Subtheme: adapting to administering insulin

In general, participants reported receiving sufficient training in administering insulin, which had usually been provided by a nurse and consisted of dummy injections. Some patients reported initial problems in terms of using the pen and others felt frightened at the idea of having to inflict daily pain on themselves:

*‘That (injecting insulin) frightened me a bit because I thought it was gonna be very…. It is gonna hurt me. In addition, I’m like…I do not like inflicting personal pain on myself’ (N01, patient, 24 months of insulin)*.

Both patients and carers also reported that skin injuries occurred as a result of transitioning to insulin. In particular, carers related their concerns to ‘old age challenges’ such as thin skin and frequent bruising:

*‘…He is over 70 years of age, and he has got quite fragile skin…he’s got loose skin and when I inject him…I have found giant lumps and …purple bruises.’ (N05, carer)*.

Most participants were far less confident about wider aspects of insulin use, such as how to adjust management while finding a stable and appropriate dose. Participants were concerned about their poor knowledge of basic topics such as interpreting glucose readings and adjusting their insulin for different meals:

*‘No, they haven’t told me, nobody told me to decrease or increase (insulin dose). It is up to me to determine’ (N08, patient, 6 months of insulin)*.

Participants explained that the general instructions provided by professionals did not take into account that the effects of insulin administration can differ across individuals. A number of participants felt, for example, that their diet recommendations should be personalised:

*‘Two people can eat the same thing. They eat differently, and it will give different results.’ (N03, patient, 36 months of insulin)*.

Some explained how they had gained weight, which had caused them significant distress, but had not been made aware of the link between weight gain and insulin use. Indeed, they were confused as to why healthcare professionals had told them to *‘keep their weight down.’ (N012, patient, 40 months of insulin)*.

Participants also reported problems with administering insulin when away from home and in particular, finding appropriate places to inject in public. Some explained that it felt stigmatising and that they were uncomfortable injecting in front of others. For example, one participant explained: *‘…the restaurant said to my husband, you’d better take her out because she’s a drug addict.’ (N012, patient, 40 months of insulin)*.

#### Subtheme: negative emotions to using insulin

Since becoming reliant on insulin, a significant worry, particularly for those who lived alone, was the risk of nocturnal hypoglycaemia. People also spoke about their fear of dying in their sleep:

*‘I was worried about…that if I go hypo at night-time and I do not wake up’ (N08, patient, 6 months of insulin)*.

While some participants were anxious about a greater reliance on their carers now they were on insulin, others were worried about their carer’s ability to recognise and manage their hypoglycaemia. Patients were often aware that carers felt nervous about administering insulin and this made it difficult for them to be confident about the process. Some carers also spoke about the responsibility of having to give daily injections:

*‘…When I inject him, sometimes he used to faint…I came maybe to realise….it is the side effects of… insulin. I’m just all alone in the house…that was a challenge for me’ (N011, carer)*.

Some older people suffered strong emotional reactions about what it meant to be placed on an insulin regimen. They explained how they felt they had *‘failed’* to take care of themselves properly and spoke of the judgements which were often attached to insulin progression by others. This had led to negative thoughts about the future, and sometimes fear that it was the end of the line in terms of their health:

*‘…It did take me quite a few months to understand, obviously, because there was a stigma in my culture…that insulin basically means that your diabetes is very uncontrolled. You’re going to die’. (N06, patient, 12 months of insulin)*

### Theme 2: what we need from healthcare services

#### Subtheme: better information about insulin

Participants wanted more information about the physiological impact of treatment, and specific advice on how to fit insulin use into their everyday lives. For example, some wanted information about how to manage insulin storage while travelling. Indeed, participants felt that there was significant amount of practical information that could improve their ongoing management, but which was not provided by healthcare professionals:

*‘These are the things that are not passed on by the professionals… it is the small things that people who could actually, um, make your life easier’ (N010, patient, 9 months of insulin)*.

In particular, participants were critical about the lack of education from primary care services, and some spoke of how doctors perceived the move from tablets to insulin as such a minor treatment change that it did not warrant providing further education:

*‘…However, because I have been having tablets all this time and just changing it to insulin, so they did not bother to explain everything.’ (N08, patient, 6 months of insulin)*.

Generally, participants were more positive about their interactions with specialist diabetes nurses, particularly those based in the hospital. Similarly, carers considered the information received in hospital to be more accurate and up to date than what they received from primary care services. Although access to these professionals could be limited, participants reported that nurses provided the most detailed and consistent supply of information about their diabetes:

*‘I usually go back to my diabetic nurse because I have had her now for four years, and we are suited for each other and she’s always there for me. I can leave her a message, and she will get back to me’. (N012, patient, 40 months of insulin)*

However, some participants were not clear about which professional had responsibility for ensuring that they were properly informed and educated. Some carers had only received information through leaflets and videos but noted that those *‘did not work for them’ (N05, carer).* Others had looked independently for further information to better understand insulin use. With limited access to in-person consultations during the pandemic, participants had relied even more on online sources. There were also concerns as to the credibility of online information and participants wished for greater signposting from professionals towards reliable and trustworthy sites.

#### Subtheme: a holistic, streamlined diabetes service

Participants related both positive and negative experiences of treatment in primary and secondary care services. Clearly, some of the comments about poor access to professionals reflected situations brought about by the COVID-19 pandemic. At this time, it was more difficult to connect to the GP for all patients and there were universal delays to specialist services. Patients experienced frustration about the brevity of their appointments with GPs. They also reported a failure to have wider discussions with their doctors about the ‘big picture’ questions such as changing their medication or coming off insulin altogether. They perceived the GP as too busy to deal with their problems and that they only offered consultations when there was a serious health issue:

*‘The doctors do not have the time to explain to you what diabetes is about and why you should be looking after it…. In addition, the consequences if you do not look after it’ (N01, patient, 24 months of insulin)*.

When moving between primary and secondary care, participants wanted a more holistic approach to treatment but felt that care providers were not working together effectively. Some were unclear where they were on the clinical pathway and about ‘*who leads my care?’ (N06, patient, 12 months of insulin)*. Participants spoke of having to see multiple different professionals who were often unaware of their previous medical history. They reported explaining personal information repeatedly due to poor communication between services. The lack of continuity was frustrating for older people:

*‘…I do not have a dedicated diabetic nurse there…you have to phone in, in the morning, they will phone you back to triage your call.’ (N10, patient, 9 months of insulin)*.

Carers also experienced inconsistent information and advice about diabetes management from health professionals across different services:

*‘The dietician said avoid excessive amounts of fruits…but the GP said you know eat more fruits and have more fibre…the GP says one thing and the hospital says another thing’ (N09, Carer)*.

Generally, patients felt reassured by their annual visits to specialist clinics for their eyes and feet in secondary care. Many believed however that the times between their clinic appointments were too long. Patients also spoke of their frustration with secondary care tests being arranged without considering what previous check-ups had occurred or noting the accessibility of service locations for patients.

### Theme 3: empowering older patients

#### Subtheme: supporting autonomy

Patients reflected on the way that doctors treated them not as a person but rather as a *‘body’ (N05, patient, 24 months of insulin)* and felt inadequately involved in their own care. Some patients explained how they had not been properly consulted in the decision to initiate insulin in the first place. Once on insulin, they spoke of insufficient detail provided by professionals to help them actively manage their diabetes care:

*‘…So when I get my blood tests, I phone up and they will say they’re just normal. However, what I like to see is actually a copy of my blood test results to make a comparison to the previous set, to monitor it.’ (N10, patient, 9 months of insulin)*.

Many wanted to discuss their individual needs with a dietician, but few had access to this. Patients felt that professionals could hold assumptions that *‘one size fits all’* in terms of diabetes care. People who were reliant on carers found it harder to connect with their GP to participate in decisions. It was noted that some services adopted a ‘*pass the parcel’ (N05, patient)* approach where patients were just told to do certain things for the sake of their health without the opportunity to question why:

*‘it should be an agreement between the person taking the insulin and the prescriber… it just appears to be…. it is a fiefdom. In addition, if you do not do as you’re told, you’re not working in this little fiefdom anymore.’ (N05, patient, 24 months of insulin)*.

#### Subtheme: do we matter?

It was commonly felt by participants that their voices and opinions were not always heard or valued. Participants mentioned that their advanced age might be the reason for a lack of interest from healthcare professionals:

*‘…You’re getting older now and you feel… They’re not interested. Cause you’re at that age where it is not exciting anymore to have to take care of you. In addition, it is a burden on the NHS….’ (N012, patient, 40 months of insulin)*.

They noticed when doctors did not provide them with enough test strips to monitor effectively or tried to get them to ration their use at home. Some felt that this was fuelled by a reluctance to spend money on older people. Other study participants were disappointed to not be offered CGM, perceiving that they were not afforded the same level of care as people with Type 1 Diabetes. Most seemed unaware that NHS services were not permitted to provide CGM for people with T2DM at that time. In addition, there were no reports of professionals suggesting to patients that they had caused their T2DM through overeating, but participants were still concerned that staff might believe this and think they were therefore less deserving of treatment.

Older people explained how they were only able to see a doctor when they experienced significant problems, and that this contributed to a feeling that their long-term health was a low priority. Some wanted regular *‘six-month consultations or checkups’ (No10, patient, 9 months on insulin*) which would reassure them that their health was important enough to be monitored. Carers also reflected on whether their relative’s age affected the level of care they received:

*‘He is 73, and part of me wonders whether or not if he was younger, then more effort will be made to help him. Because he is 73 and he’s had diabetes for quite a while…maybe it’s just something they think, well you know, his pancreas has had enough and this is kind of the usual course….” (No13, Carer).*

## Discussion and conclusion

### Discussion

This study aimed to understand the experiences of using insulin in older people with T2DM. Previous research [10, 24] has suggested that older people suffer from issues with dexterity, visual acuity and bruising. However, the older people in this study were relatively comfortable with insulin administration after appropriate training with a diabetes or practice nurse. It is difficult to assess how similar our participants were to older people who have been recruited in previous research. However, the fact that our sample was recruited online via social media may have led to obtaining participants with a relatively high level of visual and cognitive functioning. The oldest participant in our sample was only 76, hence, much older, frailer people were not represented in our study.

Despite this, our study supports previous research with type 2 patients who highlight their initial anxiety about the process of injecting [25, 26] and worries about the risk of hypoglycaemic episodes [27, 28]. In addition, participants in this study found that their adjustment to insulin use was sometimes made more difficult by feelings of ‘failure.’ These strong negative emotions attached to initiating insulin have been reported in the literature previously, particularly the perception that a move to insulin indicates that their condition has seriously deteriorated [28–30]. This study and others highlight the need to be mindful of the emotional impact that initiating insulin therapy can have on older adults [31, 32]. Staff can help to mitigate self-blame or feelings of failure in older people by better educating them about the progressive deterioration process of diabetes and emphasising the benefits of insulin to their health.

Research has shown that frequent glucose monitoring and setting appropriate glucose ranges can improve outcomes in older people with diabetes, both in terms of complications and insulin-related risks such as hypoglycaemia [7, 33]. The frustration of participants in this study regarding lack of access to specialists (e.g. dieticians) largely reflects the way that standard diabetes care is organised in the UK. Currently T2DM care falls within primary care teams although more complex cases, such as multiple daily insulin management or people with diabetes related complications, can be provided by intermediate care teams. However, study participants also spoke about the failure of primary care services to provide them with sufficient self-management resources. They cited inadequately tailored information and delayed test results, coupled with insufficient provision of strips to support monitoring. These barriers to active involvement are of concern given that diabetes self-management can improve patients’ glycaemic control, insulin adherence and disease understanding [34]. Older people were also frustrated that they were not given the opportunity to use technology such as CGM (‘flash’). In fact, such technology was not available to individuals with T2DM through the NHS at the time of the study [35, 36]. Although still not routinely provided, CGM has recently become available to people with T2DM on insulin in the UK where there is a high risk of hypoglycaemia or to address sub-optimal glucose levels in those who are able and motivated to use the technology [17].

Our participants’ emotional reactions to using insulin concur with the findings of previous studies suggesting that type 2 patients can feel disempowered, frustrated and anxious [37]. In our study, there was a perception that professionals were disinterested in optimising their health because of their age and because they had type 2 rather than type 1 diabetes. Whilst older people in our study wanted more time with their GPs to properly discuss their treatment, some thought that they were considered a drain on health service resources. Although no study participant reported being told any of this explicitly by their care team, counteracting the development of any such perceptions in older people is still important. Previous research has indicated that feelings of being undervalued are linked to a perception of self-worth in older people [13, 38]. Similarly, feelings of resignation and a lack of motivation to manage their diabetes effectively are common issues with older populations [10].

Continuity of care is a focus of health policy for people with chronic conditions such as diabetes mellitus [39]. The goal is to provide an ongoing relationship between patients and clinicians (relational continuity) and a seamless service with optimal coordination between different service providers and professionals (management continuity). A Kings Fund report [40] highlighted the importance of care coordination based on people’s individual needs and that interprofessional collaboration was necessary to ensure successful implementation. A number of participants reported that care felt like ‘pass the parcel’, i.e., they felt as if they were being passed around like a package between services who failed to communicate with each other. Participants perceived that this lack of collaborative working between primary and secondary services caused practical issues with accessing care and disparities in the advice they received.

Participants were keen for professionals to adopt a personalised approach to their treatment which considered factors related to their circumstances and age. Others explained how they did not feel comfortable enough with professionals to discuss potential changes to their regimen or management. This is again concerning, as previous research confirms that older people value being informed about decisions related to their care, and that information makes them feel significant and empowered to participate [13, 38]. Indeed, effective communication and shared decision-making have been shown to improve health outcomes and quality of life for both older people and their carers [41]. A recent codesign research workshop with older people with diabetes also highlighted the importance of professionals listening to patients to enable participation in their own care [36].

It is important to consider limitations in relation to the study sample. Late participant withdrawals led to a failure to reach our recruitment target. This reflected our difficulties in retaining older people who were already burdened with frailty and multiple health issues. Equally, with only four carers recruited, their perspectives on the experiences of older people with T2DM on insulin therapy may not fully represent those of the broader population of carers. However, despite this, we observed a significant degree of consensus between older people and carers in our sample, with similar experiences and concerns about insulin use recurring across interviews. Whilst the small sample size may have possibly affected the attainment of data saturation, many of our findings are consistent with previous research on the perspectives of older people regarding insulin initiation and management [10]. Finally, the smaller sample size is still consistent with sample recommendations for exploratory investigations of patient experiences of a specific aspect of care [21].

There are some other factors that may have led to bias in our sample. The pandemic restrictions forced us to pursue online recruitment through social media. This introduced potential selection bias towards including people who are more confident in using technology. Advertising on diabetes information support groups may have also led to the recruitment of individuals who were more engaged with their own health. Thus, our sample may have differed considerably in frailty and interest to a sample recruited in-person from healthcare clinics. Consequently, our results may overestimate the level of interest among older people in actively participating in their care and using new self-monitoring technology.

Although online recruitment made it difficult to purposively maximise variation in the sample, recruiting from Diabetes UK groups across the country rather than at local clinics ensured that older people from a wider range of geographical locations participated. We recognise however, that our findings may have limited generalisability outside of the UK in countries where the organisation and resourcing of services for diabetes are different. In terms of our sample, we chose not to record information about the ethnicity, medication use and prevalence of multimorbidity of our sample, which may have added to our understanding of insulin issues. There were notably more older men than women; hence, the views of women with T2DM may not be fully represented in the insulin experiences we considered. Equally, all the carers in our sample were female, which reflects the fact that more carers in the UK are women [42]. However, male carers may view their relatives’ health issues differently, and further research should confirm whether they require different caring support.

Finally, whilst we understood the importance of giving older people a choice of how they participated, COVID-19 restrictions at the time meant that in-person interviewing was not a possibility. Despite being offered telephone interviews, all participants opted to use online platforms. Even though orientation and technology support were provided prior to the interviews, a minority of people encountered technical difficulties. However, the interviewer attempted to minimise the impact of these issues in the process of developing rapport. In addition, the interviewer looked for important nonverbal signs that the participant was tired, uncomfortable or was becoming emotional. In these circumstances, the interviewer would change topics if they preferred or offer them the opportunity to rest or to reschedule the interview. Thus, despite minor challenges, the online interviews provided the research team with detailed information that gave valuable insights into how older people view their insulin treatment. Our experience concurs with previous researchers’ accounts of successfully interviewing older people on online platforms [43, 44].

### Practice implications

Our study suggests that some older people would welcome better information and more time in consultations to discuss diet and insulin management. Healthcare professionals should look to empower older people by providing the opportunity to discuss their blood glucose management in a more collaborative way. One strategic approach to increasing the support that people can access involves investing in the upskilling of nurses, particularly in proven techniques such as motivational interviewing [45, 46]. By enhancing nurses’ proficiency in such techniques, they can play a more significant role in patient engagement. It is also important that older people are prescribed an appropriate number of blood glucose testing strips for effective self-management. Further consideration could also be given to the benefits of trialling third-party, remote monitoring of blood glucose using continuous glucose monitoring with those patients who are interested. Older people will, of course, have different cognitive abilities and varying functional and emotional capacities for engaging with self-management and new technology. This only underlines the importance of ensuring that any plans are codeveloped and recognise the unique needs and values of the older person.

Staff need to be cognisant of the physical and emotional impact that initiating insulin therapy can have on older people. They should attempt to sensitively address feelings of blame or fear about transitioning. Providing access to peer support groups of older people may also be helpful, with research suggesting that such groups can both enhance people’s understanding of self-care and support their psychological and emotional well-being [47–49]. Healthcare professionals can play a crucial role in organising and directing older people to alternative forms of support beyond traditional doctor-centred care. The findings highlight a desire from patients for greater access to specialist advice and better collaboration between services. Addressing this could involve the implementation of a care coordination platform to ensure the accessibility of pertinent patient information for all healthcare professionals involved in their care [50]. 

Currently, research on older people with diabetes mainly focuses on the optimisation of clinical outcomes such as glycaemic levels and complications [18]. However, more empirical exploration is needed of the experiences and preferences of older people to improve their quality of life and well-being. Such research should consider the potential of training interventions for health professionals, and the co-development of educational and support services with older people to ensure that they are tailored to their specific needs and preferences [51, 52]. Given their resource implications, it is important that novel interventions are evaluated. However, the evaluation criteria should include psychosocial measures, in addition to clinical and cost outcomes, which may be more meaningful to older people [53]. Additionally, there is a need to include frailer older people who are unable to advocate for themselves and who are often excluded from research [13]. Involving family members, caregivers, and community organisations will help to include their voice in research studies. Interestingly, in this study, participants were not keen to include their carers in interviews. We did not ask for participants to justify their decision on this, but we can speculate that they may have considered themselves to be largely independent and therefore felt their carers would not have added significant information.

Our study was not focused on the needs of carers specifically, but our small sample of carers revealed their own challenges with administering insulin and hypoglycaemia fears. There is a large body of literature that points to the burden of caring for people with long-term conditions such as diabetes [54, 55], yet there is still some way to go in terms of making carers feel more confident, empowered and skilled [56]. There is clearly a need for developing education designed specifically for the carers of older people to enhance their ability to deliver effective diabetes care to their relatives. Better understanding is also required to establish how best to support the emotional needs of carers in order to reduce the negative impact that caring can have on families.

### Conclusion

The study’s findings suggest that older people may hold negative attitudes towards transitioning to insulin and can feel that healthcare services do not prioritise their health. Providing appropriate opportunities for people to actively participate in their treatment decisions and learn how to effectively self-manage their diabetes is paramount. People who care for older people also need to be included, informed and considered in such decisions where appropriate. The importance of recognising the diverse cognitive, physical, and emotional needs of older people is necessary to ensure that optimal levels of education, motivation and support are provided for each individual. This study serves as a starting point for future, larger-scale research which can confirm and extend these findings.

### Electronic supplementary material

Below is the link to the electronic supplementary material.

Supplementary Material 1

## Data Availability

All data generated or analysed during this study are included in this published article and its supplementary information files.
