# Supplementary material for: A qualitative study of the experiences of insulin use by older people with type 2 diabetes mellitus
Source: BMC Prim Care. 2024 May 22;25:180. doi: 10.1186/s12875-024-02318-3 (PMC11110346; doi:10.1186/s12875-024-02318-3)
Supplement: Supplementary file 1 — Supplementary Material 1 [file 12875_2024_2318_MOESM1_ESM.pdf]

Topic Guide

1. Reasons for referral to diabetes specialist team

Probe – explore background – had there been a crisis eg turning up at A & E?

2. Readiness to initiate insulin

Probe – had the patient been prepared and given sufficient information?

3. Need for insulin, barriers to uptake and adherence

Probe – any problems with initial prescribing?

4. Benefits of insulin

Probe – benefits of measuring doses?

5. Disadvantages of insulin

Probe – explore difficulties re administration?

6. Delay in insulin?

Probe – difficulty obtaining from pharmacist?

7. Concerns/worries before insulin initiation

Probe – was info clear? Did you feel reassured?

8. Concerns/worries during insulin initiation

Probe – were you given opportunity to raise concerns?

9. Concerns/worries during on-going care support

Probe – given info re what to expect in future?

10, Positive and negative views on insulin self-management support

Probe – good access to nurse, dietician etc?

11. Positive and negative aspects of follow-up support

Probe – good access to diabetes clinics?

12. Recommendations for future insulin initiation and on-going care support

Probe – were you given info re reduction/increase in doses?
